# De Novo Profiling of Long Non-Coding RNAs Involved in MC-LR-Induced Liver Injury in Whitefish: Discovery and Perspectives

**DOI:** 10.3390/ijms22020941

**Published:** 2021-01-19

**Authors:** Maciej Florczyk, Paweł Brzuzan, Maciej Woźny

**Affiliations:** Department of Environmental Biotechnology, Faculty of Geoengineering, University of Warmia and Mazury in Olsztyn, ul. Słoneczna 45G, 10-709 Olsztyn, Poland; brzuzan@uwm.edu.pl (P.B.); maciej.wozny@uwm.edu.pl (M.W.)

**Keywords:** lncRNAs, autonomous 3′UTRs, de novo, MALAT1, non-coding RNAs, ceRNAs, drug-induced liver injury, biomarker, liver transcriptome

## Abstract

Microcystin-LR (MC-LR) is a potent hepatotoxin for which a substantial gap in knowledge persists regarding the underlying molecular mechanisms of liver toxicity and injury. Although long non-coding RNAs (lncRNAs) have been extensively studied in model organisms, our knowledge concerning the role of lncRNAs in liver injury is limited. Given that lncRNAs show low levels of sequence conservation, their role becomes even more unclear in non-model organisms without an annotated genome, like whitefish (*Coregonus lavaretus*). The objective of this study was to discover and profile aberrantly expressed polyadenylated lncRNAs that are involved in MC-LR-induced liver injury in whitefish. Using RNA sequencing (RNA-Seq) data, we de novo assembled a high-quality whitefish liver transcriptome. This enabled us to find 94 differentially expressed (DE) putative evolutionary conserved lncRNAs, such as MALAT1, HOTTIP, HOTAIR or HULC, and 4429 DE putative novel whitefish lncRNAs, which differed from annotated protein-coding transcripts (PCTs) in terms of minimum free energy, guanine-cytosine (GC) base-pair content and length. Additionally, we identified DE non-coding transcripts that might be 3′ autonomous untranslated regions (3′UTRs) of mRNAs. We found both evolutionary conserved lncRNAs as well as novel whitefish lncRNAs that could serve as biomarkers of liver injury.

## 1. Introduction

A substantial gap in knowledge persists regarding the role of microcystins (MCs) in the underlying molecular mechanisms of organ toxicity and injury. MCs are a group of cyclic heptapeptide hepatotoxins, of which microcystin-LR (MC-LR) is one of the most widely distributed and potent variants. MC-LR is absorbed, transported and accumulated predominantly in liver [1], and it causes drug-induced liver injury (DILI). Studies on the transcriptomic level have revealed various protein coding transcripts (PCTs) involved in the response and progression of MC-LR-induced liver injury in different species [2,3]. In addition to PCTs, various non-coding RNA transcripts (ncRNAs, NCTs) have been implicated in the responses to various stressors, including DILI [4]. MC-LR alters the expression levels of small regulatory ncRNAs (shorter than 200 nt) like microRNAs (miRNAs), piwi-associated RNAs (piRNAs) and small interfering RNAs (siRNAs) [5] in various types of tissues and cells [6,7].

In comparison to knowledge about small regulatory ncRNAs, our understanding of the functions and mechanism of action of long non-coding RNAs (longer than 200 nt; lncRNAs) is still limited. Unlike miRNAs, lncRNAs are poorly conserved among species [8], which hinders research on their function and evolution. However, it has been shown that lncRNAs are involved in a variety of biological processes such as cell proliferation, apoptosis and differentiation [9] by regulating gene expression via a variety of mechanisms, including binding (sponging) miRNAs. In sponging, lncRNA competitively binds to miRNA, resulting in changes in the protein level of coding genes at the post-transcriptional level [10]. LncRNAs may function as competing endogenous RNAs (ceRNAs) that share common miRNA-response elements with PCTs [11]. For example, the recently characterized metastasis-associated-in-lung-adenocarcinoma transcript-1 (MALAT1), a well-conserved lncRNA that is implicated in diseases in humans, was shown to bind MiR34a in melanoma cells, thereby lowering MiR34a levels [12]. However, the role of MALAT1 in DILI has not yet been elucidated, and in general, our knowledge concerning the role of lncRNAs in DILI is still limited even in mammals [13].

Successful applications of RNA sequencing (RNA-Seq) technology for resolving problems pertinent to fish biology and immunology prompted us to use RNA-based methods to investigate patterns of MC-LR-induced liver injury in a teleost fish, the whitefish (*Coregonus lavaretus*). Our previous results showed that repeated exposure of whitefish to MC-LR results in severe liver damage, followed by an unexpected resilience to further exposures to the toxin and regeneration of the damaged liver structure [14]. We showed that in these adaptations, MC-LR regulates several hepatic miRNA signaling pathways and alters the expression profiles of miRNAs over the short-term [15] and long-term [16], suggesting extensive transcriptome rebuilding during these processes. Because lncRNAs have been implicated in species-specific adaptations (e.g., adaptation of zebrafish to cold [17]), a similar adaptation to MC-LR exposure that involved novel lncRNAs may have occurred in the whitefish.

Biologically active lncRNAs are present in zebrafish [18] and rainbow trout [19,20], however, these are species with well-established annotated genomes, and reference genome is not available for the majority of species. In the absence of an annotated genome, separating NCTs from PCTs requires a more challenging bioinformatic approach. As the foundation for our approach to profile lncRNAs in MC-LR-induced liver injury, we de novo assembled a whitefish liver transcriptome. Using a step-by-step pipeline designed to filter out redundant contigs, we were able to identify transcripts without coding potential, which were differentially expressed in whitefish liver after exposure to MC-LR. We identified a list of putative lncRNA transcripts, both novel and orthologous to known lncRNAs in other species. In addition, we showed that treatment of fish with MC-LR may affect levels of non-coding transcripts that may be 3′ autonomous untranslated regions of mRNAs. Furthermore, by showing how MC-LR changes expression patterns of putative lncRNAs, including MALAT1, we extended our knowledge regarding the underlying mechanisms of liver injury in whitefish. Our findings contribute to better understanding of the role of ncRNA in the molecular response to MC-LR-induced liver injury in fish.

## 2. Results

### 2.1. Sequencing Results

The number of raw reads in samples ranged from 50,797,688 to 70,885,836. The effective rate ranged from 95.88 to 99.25% ([Clean reads/Raw reads] × 100%), with a stable base error rate at 0.03% in all samples. Content of GC base pairs ranged from 47.23 to 50.50%. Detailed statistics on the quality of sequencing data for each sample are presented in Supplementary Table S1.

### 2.2. Assessing the Quality of the De Novo Assembled Liver Transcriptome

The number of detected transcripts in our raw de novo assembled liver transcriptome was 1,136,890, with an average length of 367 base pairs. After the assembly, we mapped the trimmed reads back to the assembled liver transcriptome. The fraction of aligned reads was between 97% and 98% per sample. The final assembly, which was used in further analyses, was obtained by filtering out too short, redundant and lowly expressed transcripts. The number of transcripts in our final assembly was 420,280 transcripts, with an average length of 594 base pairs. The detailed statistics of the final assembly are presented in Supplementary Table S2.

The Benchmarking Universal Single-Copy Orthologs (BUSCO) analysis pipeline revealed that of the 3640 Actinopterygian single-copy orthologs searched, our final assembly completely recovered 74.9% and partially recovered 7.5% (Table 1), while 17.6% of the single-copy orthologs were reported missing from our liver transcriptome.

### 2.3. De Novo Transcriptome Assembly Allowed Discovery and Classification of Non-Coding RNAs in Whitefish Exposed to MC-LR

**Figure 1:**
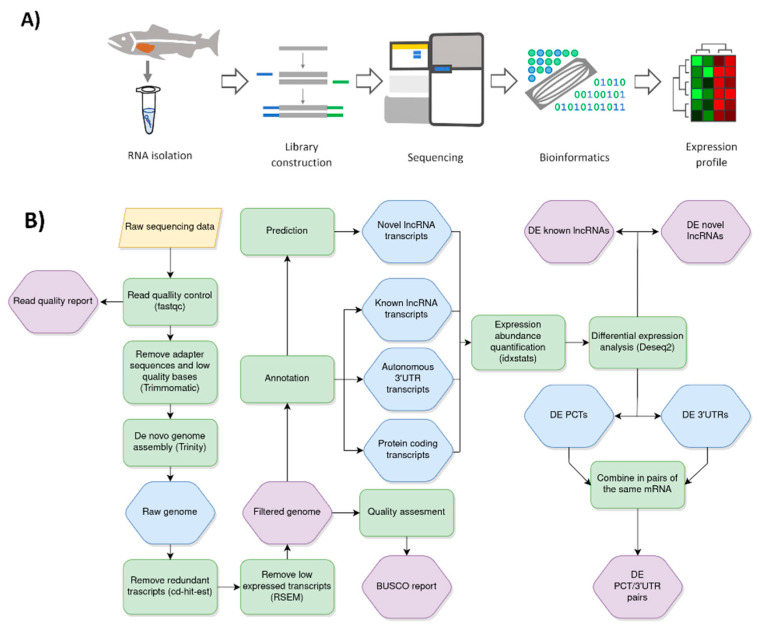
Schematic representation of pipeline used to profile changes in expression of putative long non-coding RNAs (lncRNAs) in microcystin-LR (MC-LR)-induced liver damage in whitefish. (**A**) Overview of the experimental procedure. (**B**) Bioinformatic analysis workflow. Yellow shapes indicate pipeline input, green shapes indicate action step taken in analysis, blue shapes indicate output of an action, purple shapes indicate final output. DE, differentially expressed; PCTs, protein coding transcripts.

Using the procedure for identifying ncRNAs in non-model species first described by Harris et al., we managed to first separate protein-coding transcripts (148,646 contigs) and then discover various long non-coding transcripts in whitefish (209,270) [22]. Subsequent filtering steps allowed us to separate these transcripts into three non-overlapping groups. The first group contained non-coding transcripts that showed homology to sequences deposited in the Rfam database (the group of known non-coding transcripts: 20,272 contigs). The second group contained NCTs that had homology with non-coding 3′ untranslated regions (3’UTR) of mRNA sequences deposited in the RefSeq database and had associated PCTs (autonomous 3′UTR/PCT group: 104,024 contigs). The third contained NCTs with no homology to any tested database (putative novel long non-coding RNAs: 84,974 contigs). Our filtering process is summarized in the workflow diagram (Figure 1B).

### 2.4. MC-LR Exposure Altered Expression of Evolutionary Conserved lncRNAs

**Figure 2:**
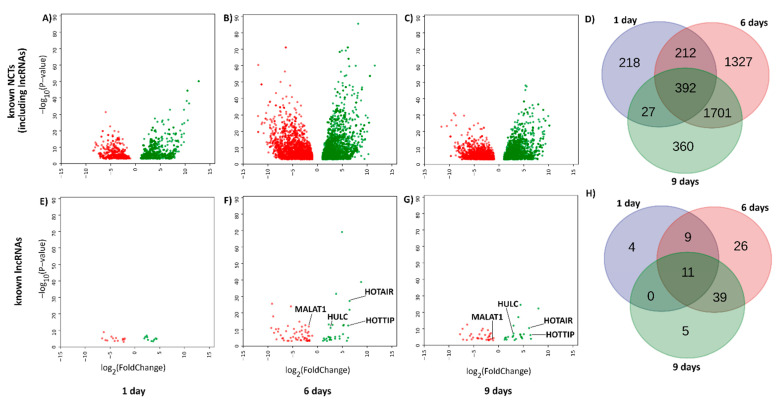
Differentially expressed putative known non-coding RNAs after MC-LR exposure. (**A**–**D**) Volcano plots and Venn diagram of differentially expressed (DE) transcripts with homology to any transcript deposited in the Rfam database (including lncRNAs). (**E**–**H**) Volcano plots and Venn diagram of DE transcripts with homology to transcripts labeled as lncRNAs in the Rfam database. Data points represent downregulated (red) or upregulated (green) putative known ncRNAs. Expression of putative metastasis-associated-in-lung-adenocarcinoma transcript-1 (MALAT1) transcript was downregulated at 6 and 9 days of microcystin-LR (MC-LR) exposure. Thresholds of a log2 fold-change > |2| and an adjusted *p*-value < 0.001 were used to filter out contigs with the smaller and less statistically significant differences between groups.

To obtain the list of evolutionary conserved lncRNAs, transcripts left after removing PCTs were additionally checked for coding potential with the support-vector machine (SVM) based Coding Potential Calculator. Non-coding transcripts were further compared with sequences of transcripts deposited in the Rfam database. This produced a list of 20,272 contigs, which were counted and analyzed for differential expression (Figure 2A–D). Among 4238 differentially expressed (DE) evolutionary conserved non-coding RNAs, there were 94 known, putatively conserved DE lncRNAs identified, including MALAT1, HOXA transcript at the distal tip (HOTTIP), HOX transcript antisense RNA (HOTAIR) and highly up-regulated in liver cancer lncRNA (HULC) (Figure 2E–H). To show similarities in sequence conservation between whitefish and 17 other species, including human, we aligned the sequence of our putative MALAT1 transcript with seed sequences of MALAT1 deposited in the Rfam database (Supplementary Figure S1).

### 2.5. Co-Expression of Autonomous 3′UTRs and Their Associated PCTs after MC-LR Exposure

**Figure 3:**
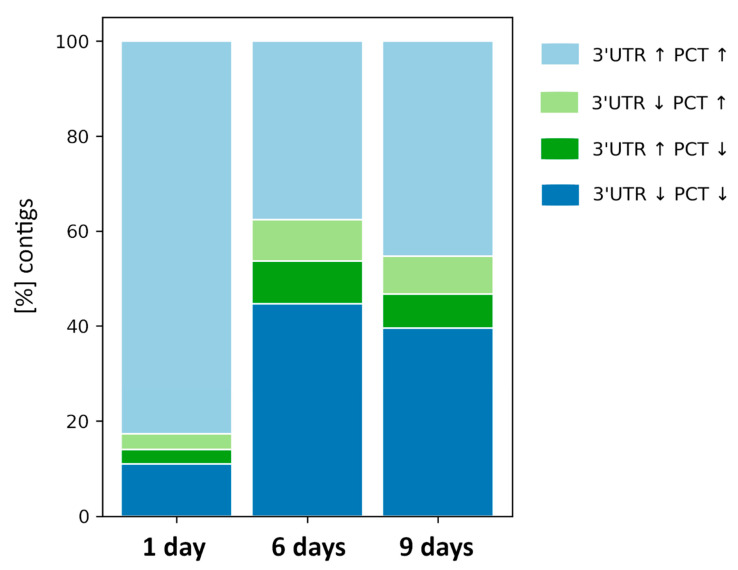
Pairs of differentially expressed (DE) putative autonomous 3′ untranslated regions (3′UTRs) and protein coding transcripts (PCTs) from the same mRNA in whitefish liver after microcystin-LR (MC-LR) exposure. Bars show percentages of DE contigs from the same mRNA that were both upregulated (light blue) or downregulated (dark blue), as well as those with opposing expression profiles (light and dark green). Arrows in the figure legend shows direction of expression changes (up- or down-regulated).

To investigate the effects of MC-LR exposure on the expression of non-coding contigs identified as autonomous 3′UTRs, we first set them together with corresponding PCTs of the same mRNA. Then, using the DESeq2 package from Bioconductor, we separately calculated the differential expression of the putative autonomous 3′UTRs and their associated PCTs. Finally, we compared the percentages of the corresponding contigs that were both up- or down-regulated, and those that were regulated in opposing directions (Figure 3). We found that, in the majority of cases, if a putative autonomous 3′UTR was DE, the associated PCT was also DE in the same direction. After 1 day of the experiment, over 82% of the transcript pairs were upregulated and only 11% were downregulated. In contrast, after 6 and 9 days, up- and down-regulated pairs were present in about the same proportions (around 40%). Moreover, we checked whether the pattern of changes in expression of co-expressed PCT/3′UTR pairs was reflected in the pattern of changes in expression of all PCTs (paired with 3′UTRs and unpaired). We found that both expression patterns were similar but not identical (data not shown).

**Figure 4:**
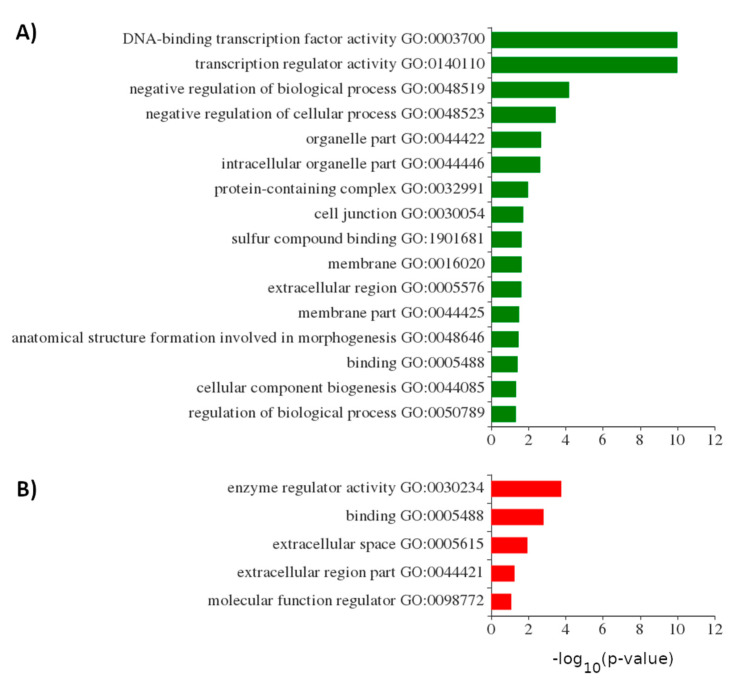
Gene ontology (GO) of co-expressed pairs of putative autonomous 3′UTRs and protein coding transcripts (PCTs) from the same mRNA that were upregulated (**A**) or downregulated (**B**) after microcystin-LR (MC-LR) exposure. Figure shows enriched (**A**) or depleted (**B**) gene ontology terms for pairs that were upregulated (**A**) or downregulated (**B**) at 1 day compared to 6 and 9 days. Horizontal bars represent the corresponding *p*-values of each regulated transcript pairs, *p* < 0.05.

Gene ontology (GO) analysis indicated similarities in terms of the pairs that were simultaneously upregulated or downregulated after 6 and 9 days of MC-LR exposure (Supplementary Figure S2). In contrast, upregulated pairs at 1 day were enriched in transcription regulator activity and DNA-binding transcription factor activity transcripts when compared with upregulated pairs at 6 and 9 days (Figure 4). Downregulated pairs at 1 day were depleted in transcripts involved in enzyme regulator activity processes when compared with downregulated pairs at 6 and 9 days of exposure. GO analysis of DE contigs from the same mRNA after 1 day of the experiment that were both upregulated or downregulated, as well as those with opposing expression profiles, are shown in detail in Supplementary Figure S3.

### 2.6. MC-LR Produced Opposing Expression Profiles of Some Autonomous 3′UTRs and Their Associated PCTs

In addition, we found that MC-LR exposure caused contrasting changes in the expression of some 3′UTRs and their associated PCTs (Figure 3, light and dark green bars). The proportion of these to all DE transcripts was lowest after 1 day of MC-LR exposure (7%), and higher at 6 and 9 days (19% and 16% accordingly). On the other hand, the proportions of both groups with the opposite expression profiles to each other remained at a similar level throughout all the days of exposure. In the group with 3′UTRs downregulated and PCTs upregulated, gene ontology terms again showed similarities on days 6 and 9, whereas on day 1, the transcripts were comparatively enriched in terms like ‘signaling’ or ‘response to stimulus’. In contrast, in the group with 3′UTRs upregulated and PTCs downregulated, transcripts involved in ‘cell’, ‘cell part’, ‘membrane’ and ‘membrane part’ were enriched on days 6 and 9.

### 2.7. Putative Novel lncRNAs and PCTs Differed in Terms Minimum Free Energy, GC Base-Pair Content and Length

**Figure 5:**
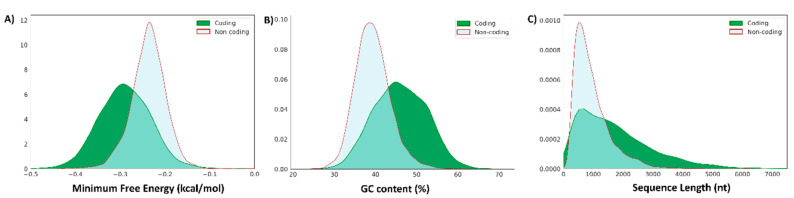
Distributions of length-corrected minimum free energy, content of guanine-cytosine (GC) base pairs and transcript lengths differ between protein coding transcripts (PCTs) and putative novel lncRNAs. (**A**) lncRNA transcripts have a higher mean length-corrected minimum free energy (−0.237 kcal/mol/nt) than PCTs (−0.289 kcal/mol/nt). (**B**) lncRNA transcripts have a lower mean GC base pair content (0.393) than PCTs (0.460). (**C**) Distribution of sequence lengths differ between lncRNAs and PCTs. Note that the distribution of PCT sequence lengths includes many longer sequence lengths.

To determine if the novel whitefish lncRNA candidates differed from PCTs in terms of a minimum free-energy (MFE), we used the RNAfold algorithm from the ViennaRNA package. The free energy values of the secondary structures were corrected for the lengths of the sequences (Figure 5A). The mean length-corrected MFE of the putative lncRNAs was significantly higher than that of the annotated protein coding transcripts (−0.237 kcal/mol/nt ± 0.038758 vs. −0.289 kcal/mol/nt ± 0.059464 kcal/mol/nt respectively, t(3999) = 46.65, *p* < 0.001, 95% [0.04963723, 0.05399174]. Moreover, the mean content of GC base pairs was significantly higher in PCTs (t(3999) = 56.16, *p* < 0.001, [0.06915164, 0.06448719]) (Figure 5B). Finally, the distribution of transcript lengths differed between the putative lncRNAs and the annotated PCTs, with more PCTs with longer sequence lengths (Figure 5C). In summary, structural differences between the putative lncRNAs and the annotated PCTs validated our methodology for discovery of lncRNAs in whitefish.

### 2.8. MC-LR Altered the Expression Profiles of the Identified Putative Novel lncRNAs

**Figure 6:**
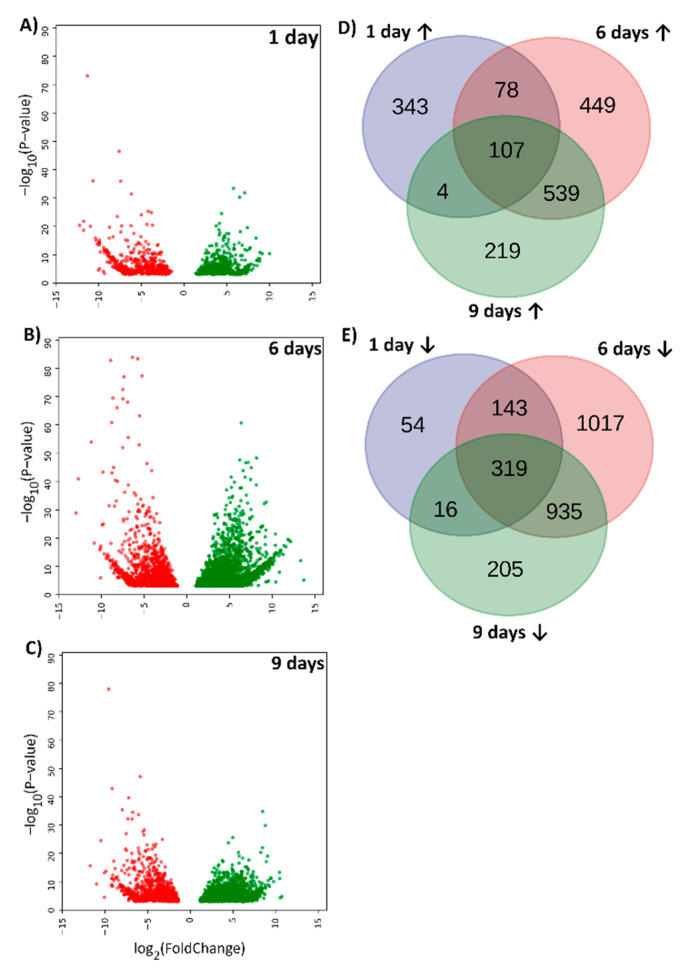
Microcystin-LR (MC-LR) induced differentially expressed (DE) putative novel lncRNAs in whitefish liver. Volcano plots after 1 day (**A**), 6 days (**B**) and 9 days (**C**) of exposure; data points represent downregulated (red) or upregulated (green) putative novel lncRNAs. Venn diagrams of upregulated (**D**) and downregulated (**E**) putative novel lncRNAs after MC-LR exposure. Note that days 6 and 9 are more similar to each other than to day 1 in terms of which lncRNAs were DE on those days. Thresholds of a log2 fold-change > |2| and an adjusted *p*-value < 0.001 were used to filter out contigs with the smaller and less statistically significant differences between groups.

Using the DESeq2 package of Bioconductor, we investigated whether MC-LR induced changes in the expression profiles of the putative novel lncRNAs discovered in this study. Using an adjusted *p*-value of 0.001 and a log2 fold-change of 2 as cutoffs, we identified 1739 and 2689 transcripts that were either up- or down-regulated by MC-LR exposure, respectively. Figure 6 shows volcano plots of up- and down-regulated putative novel lncRNAs (Figure 6A–C) and Venn diagrams with number of transcripts specific to each time period, as well as those which overlap between days of exposure to MC-LR (Figure 6D,E). In terms of changes in the expression of the identified transcripts, day 6 and day 9 were more similar to each other than to day 1: 31.0% and 34.8% of all up- and down-regulated lncRNA transcripts, respectively, were downregulated on both day 6 and day 9, but not on day 1 of the exposure (Figure 6D,E).

### 2.9. Real-Time polymerase chain reaction (RT-PCR) Confirmed Aberrant Expression of Selected Transcripts

**Figure 7:**
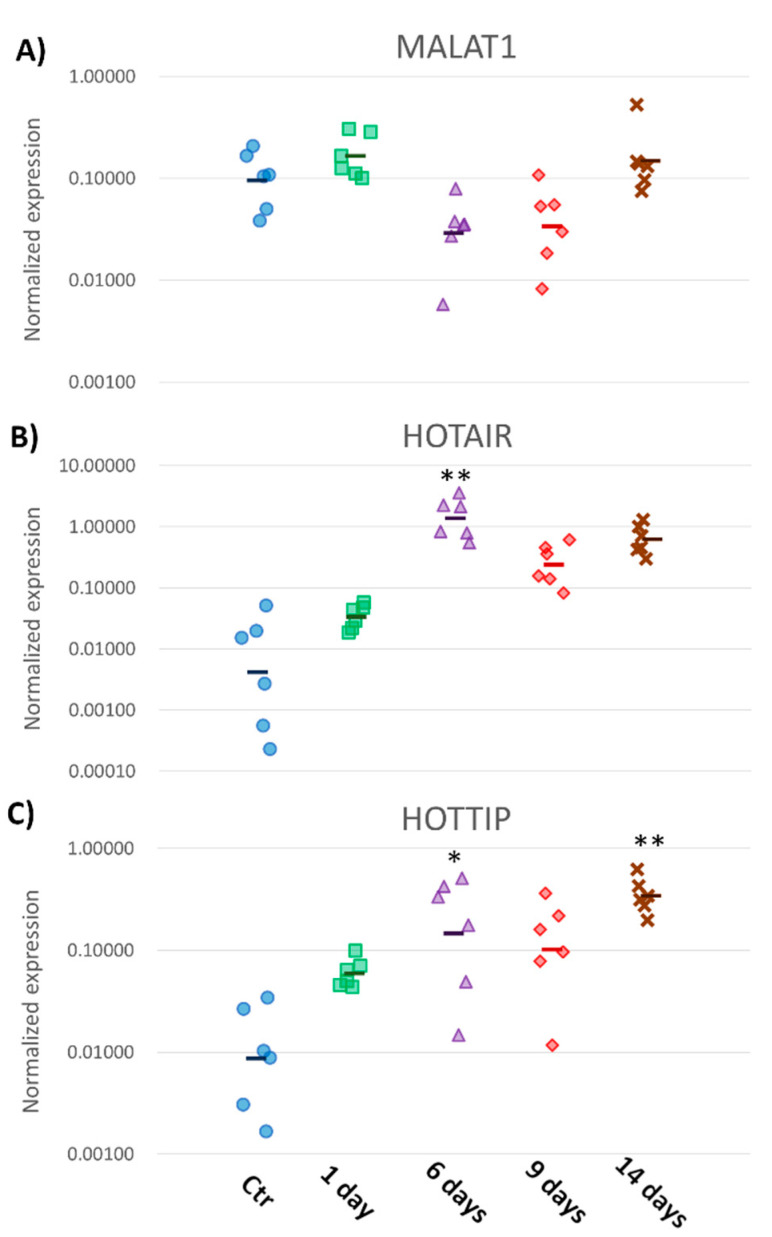
Expression of putative metastasis-associated-in-lung-adenocarcinoma transcript-1 (MALAT1) (**A**), HOX transcript antisense RNA (HOTAIR) (**B**) and HOXA transcript at the distal tip (HOTTIP) (**C**) in whitefish liver after 1, 6, 9 and 14 days of MC-LR exposure quantified using Real-Time polymerase chain reaction (RT-PCR). * *p* < 0.05; ** *p* < 0.01. Ctr—control, unchallenged group. Points represent individual fish in respective experimental group.

To validate the RNA-Seq data, we selected three known lncRNAs (Figure 7) and 10 putative novel lncRNAs (five upregulated and five downregulated, Supplementary Figure S4) and designed a RT-PCR study that re-analyzed their levels. The qPCR data indicated statistically significant changes in the expression of all selected transcripts, except MALAT1 transcripts.

## 3. Discussion

In this study, using RNA-Seq data, we identified a list of putative lncRNA transcripts involved in MC-LR-induced liver injury in whitefish, a non-model species without a reference genome. Further qPCR validation of selected putative lncRNA candidates confirmed the participation of these transcripts in MC-LR-induced liver injury in whitefish. We showed that the altered expression profiles of lncRNAs could serve as potential biomarkers of liver injury in whitefish.

The lack of standardized methodologies for discovery of lncRNAs poses a challenge for the analysis and interpretation of RNA-sequencing data. The outcome of pipelines designed to discover lncRNAs in RNA-Seq data strongly depends on factors which precede in silico analysis, such as RNA isolation or the method of preparing sequencing libraries [23]. Therefore, methods designed for enriching polyadenylated protein-coding mRNAs may not be optimal for recovering lncRNAs that are present at low levels. On the other hand, designing a large-scale RNA-Seq experiment with a large set of samples is demanding and usually some trade-offs must be made [24]. Because the main aim of our RNA-Seq experiment was to analyze the profiles of protein-coding genes, Illumina’s TruSeq Stranded mRNA protocol was chosen (Brzuzan et al., in preparation). However, this protocol can also be used for lncRNA discovery [23]. In fact, the majority of biologically functional lncRNAs reported to date are polyadenylated [25], thus approaches based on enriching polyadenylated transcripts to discover functional lncRNAs are common in pipelines based on annotated genomes, as well as those based on de novo assembled transcriptomes. For example 54,503 putative lncRNAs were discovered in rainbow trout [19] and 122,969 putative lncRNAs were reported in *Rhinella arenarum* [26]. Our pipeline based on the de novo assembly of the liver transcriptome allowed us to obtain 209,270 non-coding transcripts longer than 200 nt, of which 84,974 were labeled as putative novel lncRNAs (see further discussion, page 14).

Difficulties in comparing results of RNA-Seq in silico analysis based on de novo assembled transcriptomes may be attributed to multiple factors. For example, poor reproducibility of de novo-based analyses [27] could come from the source of the reference genome (i.e., selection of tissues), in addition to the methodologies used in preparation of the sequencing libraries. Last but not least, to obtain reliable and comparable results in discovery of lncRNAs, sequencing depth should be considered. It is estimated that, in human samples, >200 million paired-end reads are required to detect the full range of transcripts, including all possible isoforms [28]. However, this number can be much lower for differential expression analyses. For example, if the expectation is that the expression of abundant transcripts changes across conditions, 36 million reads per sample may be sufficient [24]. Because it was expected that (i) MC-LR will drastically change expression profiles of transcripts [16] and (ii) polyadenylated lncRNAs are expressed at higher abundances than non-polyadenylated lncRNAs [29], we sequenced our liver samples with 50 million reads per sample, which was sufficient to identify evolutionary conserved and novel transcripts.

In organisms without a conclusively proven reference genome, good-quality de novo assembled transcripts are a prerequisite for obtaining meaningful results in downstream analysis, such as discovery of novel transcripts [30]. We assembled whitefish liver transcriptome from 52 liver samples that originated from different experimental groups, including some that were part of another study, which extended the scope of available transcripts used for the assembly (Brzuzan et al., in preparation, Supplementary Table S1). BUSCO analysis, which estimates assembly quality based on evolutionary-informed expectations of gene content from orthologues selected from OrthoDB, showed 74.9% completeness of Actinopterygian core genes (OrthoDB v10). For comparison, the current best assembly of a whitefish full-length transcriptome based on a whole fish homogenate showed only slightly higher completeness (76.6%, OrthoDB v10, Table 1) [21]. Importantly, BUSCO recovery estimates tend to be higher in full organism assemblies than in those assembled from a set of separate tissues. For example, a whitefish tissue-based full-length transcriptome deposited in the PhyloFish database showed only 26% completeness [21]. Because our assembly of a whitefish liver transcriptome showed completeness similar to the current best whitefish whole transcriptome assemblies, we believe that it not only provided a solid foundation for our analysis, but it could also extend the completeness of current and future assemblies of whitefish transcriptomes.

Designing an accurate step-by-step pipeline for discovery of lncRNAs is an essential step for producing high-quality results. This is even more important for non-model species without a reference genome, as redundancy tends to be higher in those analyses. As a consensus state-of-the-art integrated pipeline does not yet exist, we decided to base our pipeline on a previously described pipeline for identification of lncRNAs in non-model species [22]. The core aim of this pipeline was to remove known PCTs and ncRNAs in a sequence of filtration steps. As a result, the pipeline predicts novel lncRNAs, then uses software that employs Support Vector Machine in an attempt to validate the novel transcripts by assessing protein-coding potential. Unfortunately, as both the pipeline and the validation process use BLAST results with varying levels of confidence, this validation is in fact only pseudo-independent, and thus the presented transcripts are predictions at best, and only experimental evidence can validate their true function [22]. However, a lack of an assessment of the sensitivity or the specificity of a pipeline is common among current studies aiming to classify novel lncRNA transcripts, particularly in non-model species without well-annotated genomes.

Here, we show that the putative lncRNAs differ substantially from the PCTs in terms of minimum length-corrected free energy, GC content and distribution of transcripts lengths (Figure 5). Minimum free energy is considered crucial for RNA secondary-structure stability [31]. Previous studies reported that lncRNAs have a higher free-energy level than PCTs [32,33,34], which is in line with our data. Moreover, previous findings showed that lncRNAs are less folded than PCTs due to the lower content of GC base pairs than PCTs [22,34], which was also reflected in our results. Additionally, previous studies, including studies of fish, have revealed that lncRNAs were overall enriched for shorter transcripts [19,22], which was confirmed in our study. Additionally, to validate the results of our pipeline, we performed a successful qPCR validation of selected putative lncRNA transcripts (Supplementary Figure S4).

Based on the similarity of our transcripts to the non-coding sequences deposited in the Rfam database, we identified putative whitefish liver lncRNAs whose expression was changed after MC-LR administration (known differentially expressed lncRNAs). We found that MC-LR altered expression of potent regulators of genes, such as HOTAIR, HOTTIP, HULC or MALAT1 (Figure 2E–H). Generally, lncRNAs are poorly conserved among species [8], but some of them, for example MALAT1, are conserved in mammals [35] as well as in other vertebrates, such as zebrafish [36], suggesting that they have important conserved biological roles. Moreover, MALAT1 is one of the most abundantly expressed lncRNAs in normal tissues [35,37], even similar in this regard to many protein-coding genes, such as glyceraldehyde 3-phosphate dehydrogenase (GAPDH) [38]. MALAT1 expression has been shown to be either upregulated (lung cancer, hepatocellular carcinoma) or downregulated (colorectal cancer, breast cancer) [37], indicating that its role is either cancer-promoting or tumor-suppressing. At the functional level, MALAT1 has been shown to bind various miRNAs, which promoted [39,40] or decreased cancer progression [41]. For example, knocking down MALAT1 in melanoma cells significantly upregulated the expression of tumor-suppressing MiR34a [42]. Interestingly, previous results demonstrated that MiR34a was upregulated in whitefish liver after MC-LR exposure [43,44].

Because our RNA-Seq and qPCR results showed that MALAT1 was present at a high level in normal whitefish liver, and most likely its expression was downregulated at 6 and 9 days after MC-LR exposure, this could indicate that decreased abundance of MALAT1 may also be linked with upregulated levels of MiR34a in whitefish liver after MC-LR exposure. Although the qPCR data on MALAT1 was not statistically conclusive, it was consistent with the RNA-Seq data, in that both techniques suggested or indicated, respectively, downregulation of this transcript 6 and 9 days after MC-LR exposure. Although it is too soon to speculate on whether and how this effect could potentially underlie the molecular mechanisms of MC-LR-induced liver injury, this finding adds to the little that is known about possible lncRNA and miRNA interactions in liver cells.

Previous research on MALAT1 variability has demonstrated that the majority of its transcripts are not polyadenylated [45]. However, it has been revealed that, in mammals, MALAT1 not only has a highly abundant short isoform that lacks a poly(A) tail, but also has a long isoform that is present at a much lower level and has a genomically encoded poly(A) tract. The longer isoform is further processed by RNAse P and RNAse Z to the shorter isoform. In this study, using a library preparation method that enriched only polyadenylated transcripts, we might have detected mainly polyadenylated MALAT1 transcripts, thus confirming the presence of that isoform in fish. To better study the biological function of these lncRNA, further research is required to investigate both the structural diversity and gene regulation of MALAT1.

After removal of PCTs and known NCTs, a large group of the remaining NCTs still mapped to the mRNAs deposited in the Reference Sequence database (RefSeq). However, after closer examination of particular BLAST hits, we noticed that the vast majority of our remaining NCTs mapped not to the coding parts of matched mRNA sequences, but to their non-coding 3′UTR regions. The presence of autonomous 3′UTR transcripts separated from their associated mRNAs has been documented in studies on mouse and human cells [46,47,48]. Because 3′UTR regions that were considered to be a part of the canonical transcripts are in fact biologically significant autonomous units participating in post-transcriptional regulation [48], we analyzed how expression of our putative autonomous 3′UTR transcripts corresponded to that of the PCT from the same mRNA. We found that, in the normal (unchallenged) condition, almost the same number of mRNAs had a significantly higher number of 3′UTR transcripts (48% of differentially expressed (DE) mRNAs) as those which had a higher number of PCTs (52% of DE mRNAs). This may indicate that, in normal whitefish liver, expression of those transcripts remains in a stable relationship. Moreover, we showed that exposure to MC-LR increased the abundance of PCTs while decreasing that of 3′UTR transcripts from the same mRNA (Figure 3). In pairs in which expression was changed after MC-LR exposure, 60% of the pairs had more PCTs than 3′UTR transcripts. In contrast, the same DE pairs showed the opposite pattern of expression in the control samples (i.e., 60% of the same pairs had more 3′UTR transcripts), indicating that MC-LR changed the PCT/3′UTR ratio in about 20% of DE mRNAs (depending on the duration of exposure). This also indicated that, after MC-LR exposure, expression of our putative 3′UTR transcripts changed independently of PCTs expression.

In the majority of cases, if a putative autonomous 3′UTR was DE after MC-LR exposure, the associated PCT was also DE in the same direction. Our results show that, out of all DE PCT/3′UTR pairs after 1 day of MC-LR exposure, over 82% of the paired transcripts were upregulated (Figure 3). In contrast, at after 6 and 9 days of exposure, there was no longer a majority of PCT/3′UTR pairs that were DE in the same direction. Moreover, gene ontology terms analysis showed that, after 1 day of exposure, the co-upregulated pairs were enriched in transcription regulators, suggesting that these transcripts play roles in regulating the response to severe liver damage (Figure 4A). For example, this group included both ATF3 and FOS, the subunits of the AP-1, a pro-apoptotic transcription factor. This is in line with recently published results of transcriptomic profiling of microcystin-LR in human hepatocyte cell line [49]. On the other hand, after 1 day of exposure, the co-downregulated pairs were depleted in enzyme regulators, including phosphatases, which is the canonical mode of action of microcystins [50] (Figure 4B). 

Additionally, our results show that DE PCT/3′UTR pairs at 6 and 9 days of exposure are similar in terms of function and direction of expression changes (Supplementary Figure S2). The observed changes in the liver transcriptome between the 1st and the 6th or 9th day of exposure may support our previous finding that challenging whitefish with MC-LR results in severe liver damage, which is followed by resilience to further exposures to the toxin, allowing for regeneration of the damaged organ [14]. It should be investigated whether this apparent shift in the transcriptome profile reflects remodeling of liver cell processes for repair of the tissue.

Moreover, we also found that, in response to MC-LR exposure, some putative autonomous 3′UTRs and PCTs from the same mRNA were differently expressed in opposite directions, i.e., one was upregulated while the other was downregulated (Figure 3, light and dark green bars). We were particularly interested in pairs where the 3′UTR transcripts were upregulated and the PCTs were downregulated, as this could be attributed to a recently discovered mechanism in which the 3′UTR is cleaved and shortened [48]. The discoverers of that mechanism hypothesized that it serves as a global regulatory tool that works by increasing the effectiveness of miRNA binding sites upstream of the cleavage site. This would cause levels of 3′UTRs to rise after cleavage, while levels of the corresponding PCTs would drop as a result of more effective miRNA binding. Previous studies showed that miRNAs that play roles in transcription regulation are also aberrantly expressed after exposure to MC-LR [5,16,51,52]. Because there is a possible crosstalk between various types of NCTs participating in post-transcriptional regulation of PCTs in MC-LR-induced liver injury, our results suggest the necessity of adopting a wider perspective when investigating the effects of MC-LR-induced liver damage. Researchers looking to discover all aspects of transcriptome regulation by ncRNAs in MC-LR toxicity should focus on decoding the crosstalk between NCTs and PCTs, i.e., competition between endogenous RNAs [53]. It has been shown recently that this strategy can be applied to successfully investigate mechanisms of MC-LR toxicity in other tissues. For example, Meng et al. showed a transcriptomic regulatory network of miRNAs, piRNAs, circular RNAs, lncRNAs and mRNAs that were simultaneously involved in the cytotoxicity of MC-LR in testicular tissues in mice [5].

Gene ontology terms analysis of pairs where the 3′UTR transcripts were upregulated and the PCTs were downregulated (Supplementary Figure S3D) showed genes involved in the catalytic activity and binding processes, particularly mapk3 (non-specific serine/threonine protein kinase) involved in mitogen activated protein kinase (MAPK) signaling. MAPK signaling is induced by MC-LR, which inhibits PP2A phosphatases [54]. Active MAPKs stimulate the synthesis and phosphorylation of transcription factors, which leads to an increase in cellular proliferation and tumor promotion [55]. Because it was shown that mapk3 is regulated by MIR550a-3p in breast cancer [56], it may be possible that increased MAPK signaling is compensated by the mechanism of shortening of 3′UTRs. Similarly, we also observed enriched transcripts involved in the metabolic pathways, for example, GTP cyclohydrolase 1 encoded by the GCH1 gene, which is responsible for the hydrolysis of guanosine triphosphate (GTP). In a recent study, GCH1 was found enriched in human hepatocellular carcinoma [57]. GCH1 gene expression is regulated post-translationally, e.g., by phosphorylation [58], but it was also shown that GCH1 mRNA is a target of miR-133a in endothelial cells [59].

Although we showed that the putative 3′UTR contigs were expressed independently of PCTs, some of the autonomous 3′UTRs paired with PCTs from the same mRNA could in fact be fragmented or incomplete mRNAs which were not assembled properly. However, even if a de novo transcriptome assembly approach for detecting various types of non-coding RNAs is not without flaws, it can be used as an extension to analyses based on annotated genomes. Because the detection of autonomous 3′UTR transcripts using an annotated genome needs additional enrichment of 3′UTR transcripts in the RNA isolation or library preparation steps, it requires additional sequencing runs. Alternatively, pipelines designed to analyze autonomous 3′UTRs based on the de novo assembled transcriptome may be used in a preliminary research, eventually leading to more focused sequencing runs. Furthermore, the huge collection of deposited transcriptomic data may be reused for additional de novo analysis by teams seeking direction for their studies on non-coding RNAs.

The aforementioned group of 84,974 putative novel lncRNAs contained transcripts longer than 200 nucleotides with no homology to any tested database. We showed that MC-LR upregulated expression levels of 1739 putative novel lncRNAs and downregulated 2690 (Figure 6). We found that MC-LR-induced change in expression of putative novel lncRNAs was also reflected in change in expression of PCTs (data not shown). Because, as for now, co-expression of lncRNAs with PCTs is the most common approach for identifying potential target genes of lncRNAs [12], this will allow to identify potential target genes of lncRNAs, and to investigate their biological function in MC-LR-induced liver injury in whitefish. Moreover, putative novel lncRNAs might also serve as potential biomarkers for early detection of severe liver injury in whitefish (1 day), as well as the fish’s recovery from exposure to the toxin, including regeneration of liver tissues (6 and 9 days). As demonstrated in certain types of cancers [60], lncRNAs have highly specific expression patterns and relatively stable secondary structures, and are efficiently detected in blood, plasma and urine. With these properties, they have the potential to serve as novel noninvasive biomarkers for drug-induced liver injury [13,61,62]. Additionally, our previous results suggests that the non-coding MiR122 can be a non-invasive biomarker for detecting liver damage in fish, and a promising alternative to current gold-standard hepatotoxicity markers [63].

The adverse effects of MC-LR in whitefish are not limited only to the liver. For example, we previously showed that MC-LR caused brain injury in whitefish [64]. Although plasma levels of brain-specific MiR124-3p were not altered, it is possible that some brain-specific lncRNAs could serve as biomarkers of brain injury. Interestingly, MALAT1 shows relatively high expression in the brain, where it is involved in regulating synaptogenesis [65]. MALAT1 was downregulated in glioma [66] and is able to regulate levels of MiR124 in various diseases, including Parkinson’s disease [67]. Future studies will advance understanding of the roles of lncRNAs in MC-LR toxicity and will likely reveal novel biomarkers and targets for treatment. It will also be important to determine whether the aberrantly expressed lncRNAs detected in this study can be detected in noninvasively collected samples.

## 4. Materials and Methods

### 4.1. Fish Maintenance and Exposure

This study is a part of larger project which aimed to examine changes in the liver transcriptome of whitefish exposed to MC-LR and the effects of intervention with the use of microRNA 92b-3p synthetic analogs (mimic and inhibitor) on the transcriptome. Experimental details concerning maintenance and exposure of the individuals used in this project will be described in separate papers (Brzuzan et al., Woźny et al., in preparation). In the current study, a total number of 52 individuals from the RNA-Seq of the whole project were used in order to assemble the reference transcriptome (Supplementary Table S1). However, only 30 individuals from the project were used to assess MC-LR effects on expression of non-coding transcripts.

Fish maintenance and exposure were conducted at the Department of Salmonid Research in Rutki (Inland Fisheries Institute in Olsztyn, Poland). The department also provided fish for this study. All animal-related procedures were approved by the Local Ethics Committee for Experiments on Animals in Olsztyn, Poland (resolution No. 44/2016 of 30 November 2016). Juvenile whitefish (mean ± standard deviation (SD): 29.9 ± 1.6 g, 17.0 ± 0.4 cm) were kept in flow-through tanks supplied with well (underground) water. Water temperature in the tanks was 9.3 ± 0.2 °C and oxygen level was 10.3 ± 0.6 mg·L^−1^. Throughout the experiment, all the fish were fed with a minimal feeding procedure dependent on the water temperature, caloric content of the feed and predicted fish mass. However, 1–2 days prior to exposure (intraperitoneal injections) or collection of samples, the fish were deprived of food.

The dose of MC-LR (100 μg·kg^−1^ of body mass) and the treatment periods (1, 6, 9 and 14 days) were based on our previous studies on molecular and physiological responses of whitefish to this toxin [14,16,68]. MC-LR (purity ≥ 95%; high-performance liquid chromatography, HPLC) was obtained from Enzo Life Sciences (Enzo Biochem, Inc.; Farmingdale, NY, USA) and dissolved in phosphate buffer saline (PBS) as a solvent vehicle. Prior to exposure, randomly selected individuals from each group were anesthetized by immersion in MS-222 solution, and then they received an intraperitoneal injection of the MC-LR solution. To maintain continuous exposure, the MC-LR injection (100 μg·kg^−1^ of body mass) was repeated after 7 days of the experiment. Fish that received an intraperitoneal injection with pure PBS served as a negative control group. Throughout the exposure period, fish from the different groups were kept in separate tanks. Each experimental group (PBS or MC-LR) in each treatment period (1, 6, 9 and 14 days) consisted of *n* = 6 individuals, thus in total, 30 individuals were used in our MC-LR-treatment study. The number of fish in each experimental group was estimated based on our previous work [16], where we demonstrate that this group size is sufficient to observe a distinct effect of MC-LR on ncRNA expression pattern.

After each exposure period, randomly selected individuals from each group were euthanized by the MS-222 anesthetic overdosing (immersion in 300 ppm solution) and fragments of their livers were collected and preserved in RNAlater solution according to the manufacturer’s recommendations (Sigma-Aldrich, St. Louis, MO, USA). All the fish used in this study were euthanized via an overdose of MS-222.

### 4.2. RNA Isolation, Sequencing and Initial De Novo Assembly

Total RNA was extracted from the RNAlater-preserved liver fragments (approximately 20 mg) using a PureLink RNA Mini Kit (Life Technologies, Carlsbad, CA, USA) according to the manufacturer’s protocol. To remove the genomic DNA residue, the extracted samples were incubated with TURBO DNAse (Invitrogen, Carlsbad, CA, USA) and then purified using the PureLink RNA Mini Kit. RNA integrity was evaluated with an Agilent Bioanalyzer 2100 with an Agilent 6000 Nano Kit and the samples with mean RNA integrity number (RIN) > 8 were taken for library preparation with the Illumina TruSeq Stranded mRNA Library Prep protocol. The libraries were sequenced with an Illumina HiSeq4000 sequencer (250–300 bp insert cDNA size, PE150, 50 M reads, 15 Gb).

The workflow used to profile changes in expression of putative ncRNAs in MC-LR-induced liver injury in whitefish is shown in Figure 1. Quality control of raw sequencing reads was performed with FastQC, version 0.11.8. To remove adapter sequences and low-quality bases, the reads were processed using Trimmomatic, version 0.36 [69]. After quality trimming, every 6th read (starting from the 6th) was selected for downstream analysis. Selected reads were assembled into a reference genome using Trinity, version 2.5.1, with the default parameters [70]. Trimmed reads were mapped back to the reference genome using Bowtie2, version 2.3.5.1 [71].

### 4.3. ncRNA Identification Pipeline

The following pipeline was based on Reference [22]. First, the Trinity de novo assembled genome was filtered for redundant transcripts using the cd-hit-est algorithm of CD-HIT [72], with a sequence identity threshold of 0.9. Filtering by expression was executed with RSEM [73] implemented by the Trinity-provided perl script ‘align_and_estimate_abundance’. Transcripts with expression levels below FPKM = 1.50 (fragments per kilobase of transcript per million mapped reads) were filtered out from the dataset.

To assess the quality of the filtered de novo assembled transcriptome, we quantified its completeness by comparing it with a set of highly conserved single-copy orthologs. Using the BUSCO v2 pipeline [74], we compared our assembly with the predefined set of 3640 Actinopterygian single-copy orthologs from the OrthoDB v10 database [75]. BUSCO analysis calculated the number of orthologs, whose length was within two standard deviations of the mean length of the given BUSCO (complete BUSCOs, C), complete BUSCOs represented by single-copy transcript (single-copy BUSCOs, S), complete BUSCOs evidenced by more than one transcript (duplicated BUSCOs, D), partially recovered BUSCOs (fragmented BUSCOs, F) and not recovered BUSCOs (missing BUSCOs, M). To verify our assembly, we repeated exactly the same procedure with the most complete whitefish whole transcriptome available to date [21].

Next, the transcripts were searched for open reading frames (ORFs) by Transdecoder, version 2.0.1 [76]. To identify protein coding transcripts, ORFs and transcripts were searched against the UniProt-Swiss-Prot and Atlantic salmon proteins reference databases (GCF_000233375.1) using blastp and blastx from the BLAST+ suite with a threshold E-value of 1 × 10^−3^ [77]. Protein family searches were performed with the Pfam 32.0 database [78] using the ORF protein sequences in HMMER, version 3.2.1. Finally, the top BLAST hit based on the bit score, E-value and percent alignment, and all HMMER hits were loaded into Trinotate, version 3.2.1, to generate an annotation report [79]. Based on the report, transcripts that were not PCTs were then filtered against the RFAM database, version 12.0 [80], by the cmscan algorithm implemented by Infernal, version 1.1.3 [81]. Any hit that Infernal considered significant using the default parameters was filtered out (and labeled as a known ncRNA). All remaining putative novel NCTs were further validated by calculating coding potential using Coding Potential Calculator (CPC) [82].

To further verify that the remaining contigs were completely separated from mRNAs, putative novel non-coding transcripts were subjected to a blastn search against the Reference RNA Sequences database (NCBI, refseq_rna). Any transcript that was identified as “mRNA” was set together in a pair with corresponding PCT of the same mRNA. Only transcripts which had a corresponding PCT were subjected to further analysis (putative autonomous 3′UTRs).

At this point, all transcripts that were considered to be either known ncRNAs or putative novel ncRNAs, as well as transcripts identified as Atlantic salmon proteins (PCTs) and putative autonomous 3′UTR transcripts, were counted in each sequenced sample using samtools idxstats (Figure 1B) [83].

### 4.4. Free-Energy Levels of Non-Coding Transcripts

The minimum free-energy of each transcript was calculated using the rnafold algorithm implemented by ViennaRNA, version 2.4.12 [84], using the following options: –p –d2 –noLP. The minimum free-energies of the transcripts were then compared to the minimum free-energies of a randomly selected set of protein coding transcripts (Figure 5A).

### 4.5. Functional Annotation of Putative Autonomous 3′UTRs and PCTs of the Same mRNA

GO analysis (http://www.geneontology.org) was performed to construct gene annotations. To retrieve GO IDs for particular proteins, we used the Retrieve/ID mapping tool from the UniProt website [85]. WEGO (Web Gene Ontology Annotation Plot) was used to visualize the results [86]. A *p*-value < 0.05 was considered to indicate a statistically significant difference.

### 4.6. Real-Time PCR

To profile known and putative novel lncRNA expression, reverse transcription (RT) was carried out using SuperScript IV Reverse Transcriptase (Thermo Scientific, Waltham, MA, USA). The reaction contained 1 µg total RNA, 4 µL 5× RT buffer, 1 µL 0.1 M DTT, 1 µL 10 mM dNTP mix, 1 µL Ribonuclease Inhibitor and SuperScript IV RT enzyme, and 1 µL 50 µM Oligo(dT)20 primer. The reaction was carried out at 23 °C for 10 min, 55 °C for 10 min and 80 °C for 10 min. Synthesized cDNA samples were diluted (20×), stored at −80 °C, and thawed only once, just before the amplification.

Real-time PCR was used to determine relative expression of known and putative novel lncRNAs in the cDNA samples. Reactions were carried out in final volumes of 20 µL, consisting of 10 µL Power SYBR Green PCR Master Mix (Life Technologies, USA), 0.25 µM of each primer (forward and reverse; Table S3), 1 µL cDNA template and 7 µL PCR-grade water. Amplification was performed with a Quant Studio 5 Real-time PCR System (Applied Biosystems; Foster City, CA, USA) with the following conditions: 95 °C for 10 min, then 45 cycles of 95 °C for 15 s and 60 °C for 1 min. The reaction for each sample was carried out in duplicate. No-template controls (NTCs) were included to test for the possibility of cross-contamination. To check the quality of each PCR product, melting curve analyses were performed after each run. For normalization of data from the treatment (MC-LR) and control (PBS) groups, “uncharacterized transcript” was used as a reference gene. This transcript was selected based on RNA-Seq results. Its stability was confirmed by Real-Time PCR (standard deviation (SD) of quantification cycle (Cq) ± 0.84).

### 4.7. Statistical Methods

Contigs that were differentially expressed (DE known and novel lncRNAs, DE 3′UTRs, DE PCTs) in the MC-LR-treated and the control (PBS) groups were indicated using the DESeq2 package, version 1.28.0 [87] for R, version 3.6.3 [88]. Adjusted *p*-values were calculated with Benjamini and Hochberg’s method [89]. Thresholds of a log2 fold-change >|2| and an adjusted *p*-value < 0.001 were used to filter out contigs with the smaller and less statistically significant differences between groups.

Before assessing the differences in minimum free-energy and content of GC base pairs, we used histograms and normal Q-Q plots (shown in Supplementary Figure S5) to assess the distribution of the data. These methods indicated that, considering the large sample size, any deviations from normality were too small to be important. Thus, we assessed differences between groups using Welch’s *t*-test, which is robust to violations of the assumption of homoscedasticity. 95% confidence intervals for differences are shown in square brackets in the main text, e.g., [9.5, 11.7]. Statistical calculations were handled in Python’s statistical modules (Sci-Py, StatsModels). Confidence intervals were calculated using the R Tidyverse package.

Statistical calculations for qPCR data were performed using GenEx 7.0. To assess the significance of difference between groups, one-way independent analysis of variance (ANOVA) was used after log-transforming the data, followed by Dunnett’s post hoc test.

### 4.8. Data Availability

The raw data from this study have been submitted to the NCBI SRA database. The accession numbers for data from the individual samples are given in Supplementary Table S1. De novo assembled whitefish liver transcriptome and sequences of transcripts identified in this study have been deposited in The Dryad Digital Repository (https://datadryad.org).

## 5. Conclusions

In this study, we detected differentially expressed polyadenylated lncRNAs in whitefish exposed to MC-LR. To achieve this, we constructed an extensive liver transcriptome that can be used to complete the current whitefish genome assemblies or to curate ones that are developed in the future. We obtained a dataset that provides a starting point for future studies on the role of lncRNAs in MC-LR-induced liver injury and subsequent liver regeneration. Among the detected DE transcripts, we identified novel, uncharacterized contigs that could potentially be used as non-invasive biomarkers of MC-LR-induced liver injury in whitefish. In addition, we detected transcripts with homology to lncRNAs previously described in other species. Current understanding of lncRNAs is limited [90], and we believe that our work is a step toward better understanding lncRNA expression and functions in the context of MC-LR toxicity, and the mechanisms of MC-LR toxicity in general. Lastly, we believe that to fully understand the molecular functions of lncRNAs, studies should adopt a broader perspective, including simultaneous analysis of all aspects of the network of competing endogenous RNAs. For that purpose, a combination of different methods of library preparation for RNA sequencing should be considered, which also may allow new types of RNA to be uncovered.

## Figures and Tables

**Table 1:** Summary of the complete, duplicated, fragmented and missing orthologs inferred from the Benchmarking Universal Single-Copy Orthologs (BUSCO) search against the orthologs for *Actinopterygii.*

BUSCO Statistic	Whitefish Liver; OrthoDBv10 (This Study)	European Whitefish Whole Transcriptome; OrthoDBv10 [21]
Complete BUSCOs (C)	2725 (74.9%)	2786 (76.6%)
Complete and single-copy BUSCOs (S)	1780 (48.9%)	1713 (47.1%)
Complete and duplicated BUSCOs (D)	945 (26.0%)	1073 (29.5%)
Fragmented BUSCOs (F)	272 (7.5%)	320 (8.8%)
Missing BUSCOs (M)	643 (17.6%)	534 (14.6%)
Total BUSCO groups searched	3640	3640

## Data Availability

The raw data from this study have been submitted to the NCBI SRA database. The accession numbers for data from the individual samples are given in Supplementary Table S1. De novo assembled whitefish liver transcriptome and sequences of transcripts identified in this study have been deposited in The Dryad Digital Repository (https://datadryad.org). The results of gene expression have been submitted to the NCBI Gene Expression Omnibus database.

## Supplementary Materials

Supplementary Materials can be found at https://www.mdpi.com/1422-0067/22/2/941/s1. Table S1: Detailed statistics on the quality of sequencing data from all (52) samples used for de novo assembly of the whitefish liver transcriptome. Samples in bold were used in this study for quantitative analysis of lncRNA. Table S2: Contig metrics of the filtered de novo assembled liver transcriptome. All metrics were calculated using Transrate, version 1.0.3. Table S3: Sequences of primers used in RT-qPCR. Figure S1: Alignment of our putative MALAT1 transcript to the seed sequence of human MALAT1 deposited in the Rfam database. (A) Output of cmscan hit alignment. (B) ClustalO alignment. Figure S2: Comparison of gene ontology terms of co-upregulated (A) and co-downregulated (B) putative PCT/3′UTR transcript pairs at 6 days (red bars) and 9 days (gray bars) of MC-LR exposure. Figure S3: Gene Ontology terms of DE contigs from the same mRNA after 1 day of the experiment that were both upregulated (A) or downregulated (C), as well as those with opposing expression profiles (B,D). Figure S4: Expression of selected putative novel lncRNAs in whitefish liver after 1, 6, 9 and 14 days of microcystin-LR (MC-LR) exposure quantified using RT-qPCR. Transcripts were selected based on RNA-Seq results. * *p* < 0.05 and ** *p* < 0.01. Points represent individual fish in respective experimental group. Figure S5: Histograms and normal Q-Q plots of minimum free-energy (MFE), content of GC base pairs and sequence length in protein-coding transcripts (PCTs) and non-coding transcripts (NCTs).

## Abbreviations

ATF3	activating transcription factor 3
BLAST	Basic local alignment search tool
BUSCO	Benchmarking set of Universal Single-Copy Orthologs
bp	base pair
DE	Differentially expressed
DILI	Drug-induced liver injury
FOS	fos proto-oncogene
GCH1	GTP cyclohydrolase 1
GO	Gene ontology
HOTAIR	HOX transcript antisense RNA
HOTTIP	HOXA transcript at the distal tip
HULC	Highly up-regulated in liver cancer
lncRNA	long non-coding RNA
MAPK	mitogen activated protein kinase
MC	microcystin
MC-LR	microcystin-LR
NCBI	National Centre for Biotechnology Information
ncRNAs	non-coding RNAs
NCTs	non-coding transcripts
miRNA	microRNA
ORF	Open Reading frame
PCTs	protein-coding transcripts
piRNA	piwi-associated RNA
RIN	RNA integrity number
RT	Reverse transcription
siRNA	small interfering RNA
SVM	Support Vector Machine
