# Supplementary figures and images for: De Novo Profiling of Long Non-Coding RNAs Involved in MC-LR-Induced Liver Injury in Whitefish: Discovery and Perspectives
Source: Int J Mol Sci. 2021 Jan 19;22(2):941. doi: 10.3390/ijms22020941 (PMC7833382; doi:10.3390/ijms22020941)

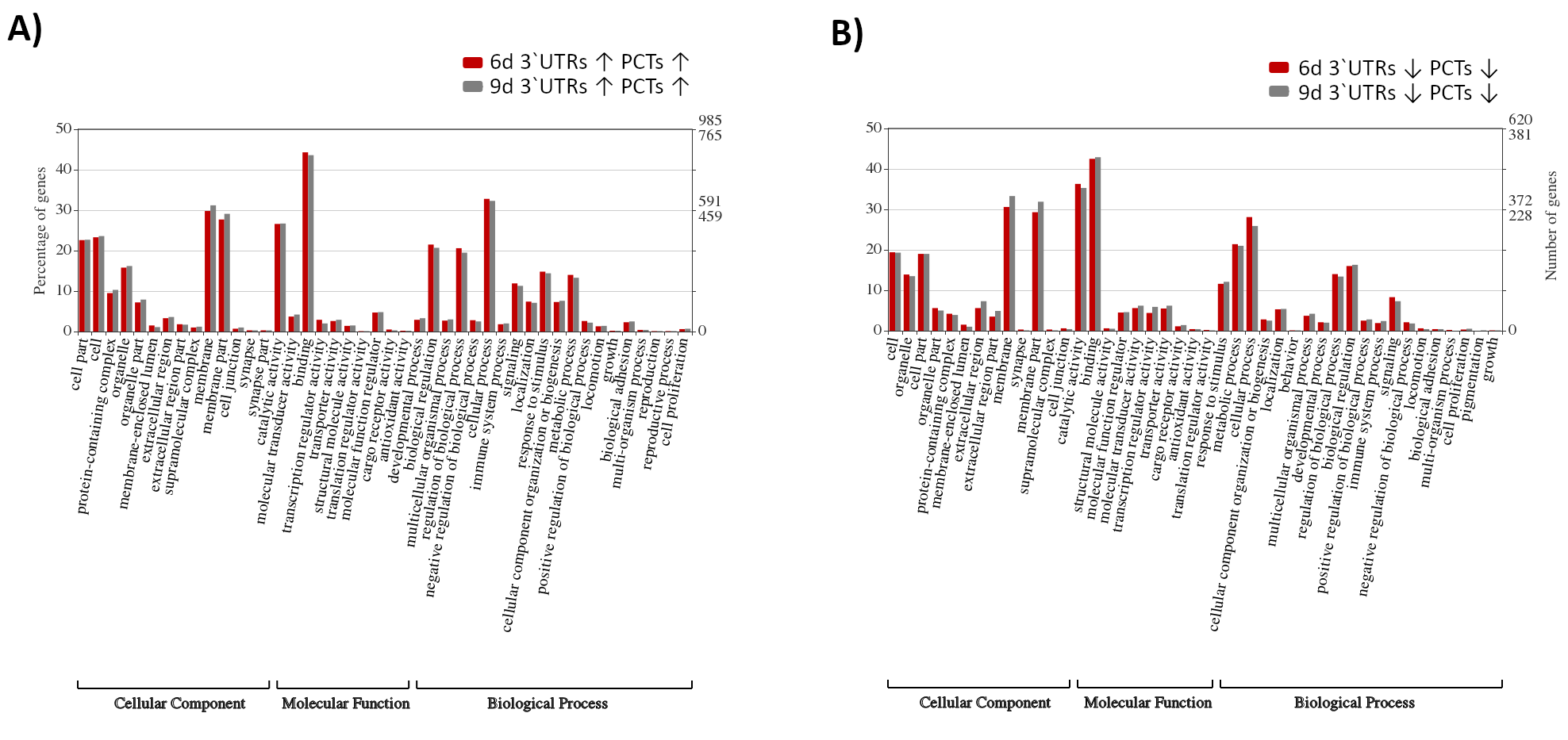

Supplement: Supplementary file 1 [file ijms-22-00941-s001.zip › Figure_S2.tif]

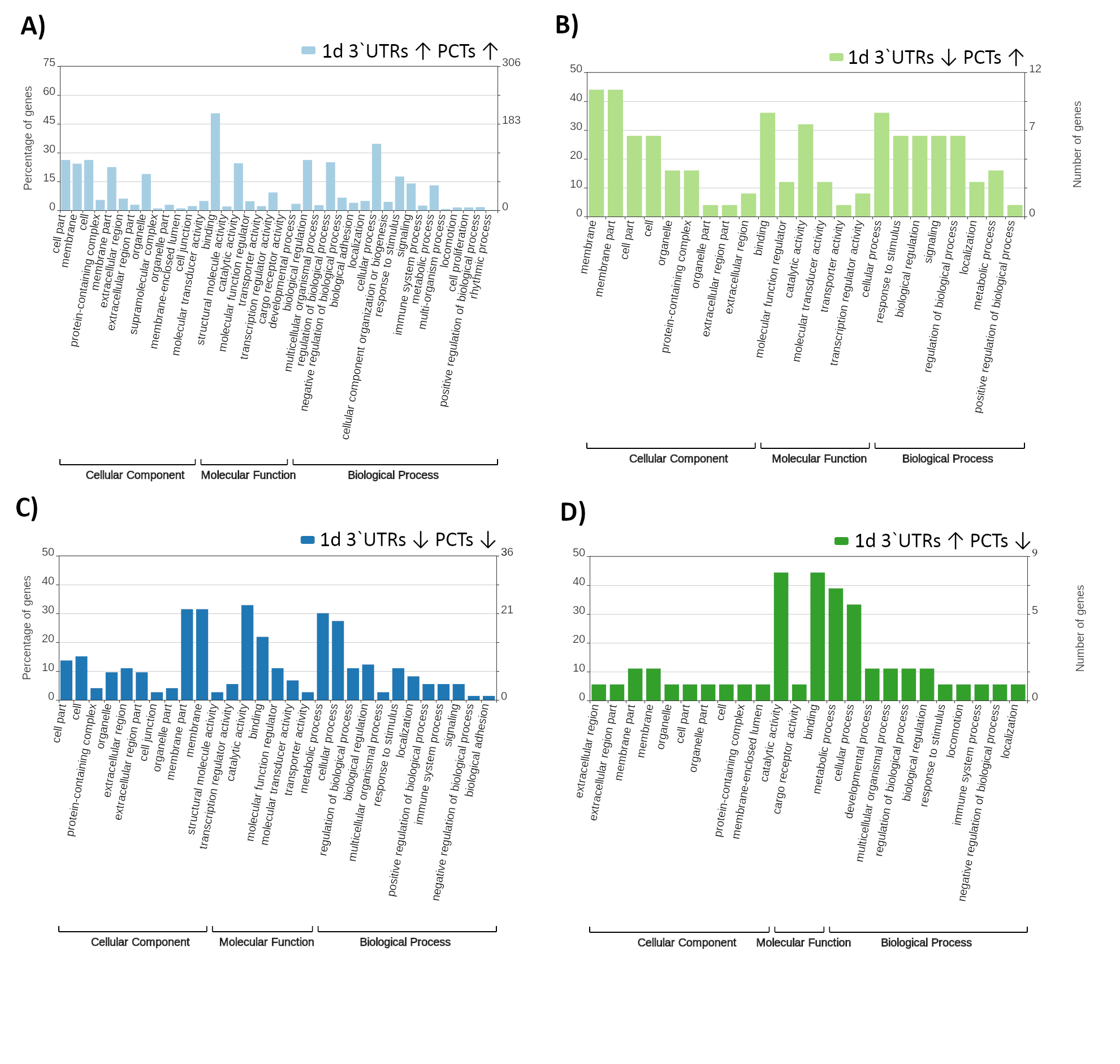

Supplement: Supplementary file 1 [file ijms-22-00941-s001.zip › Figure_S3_v2.tif]

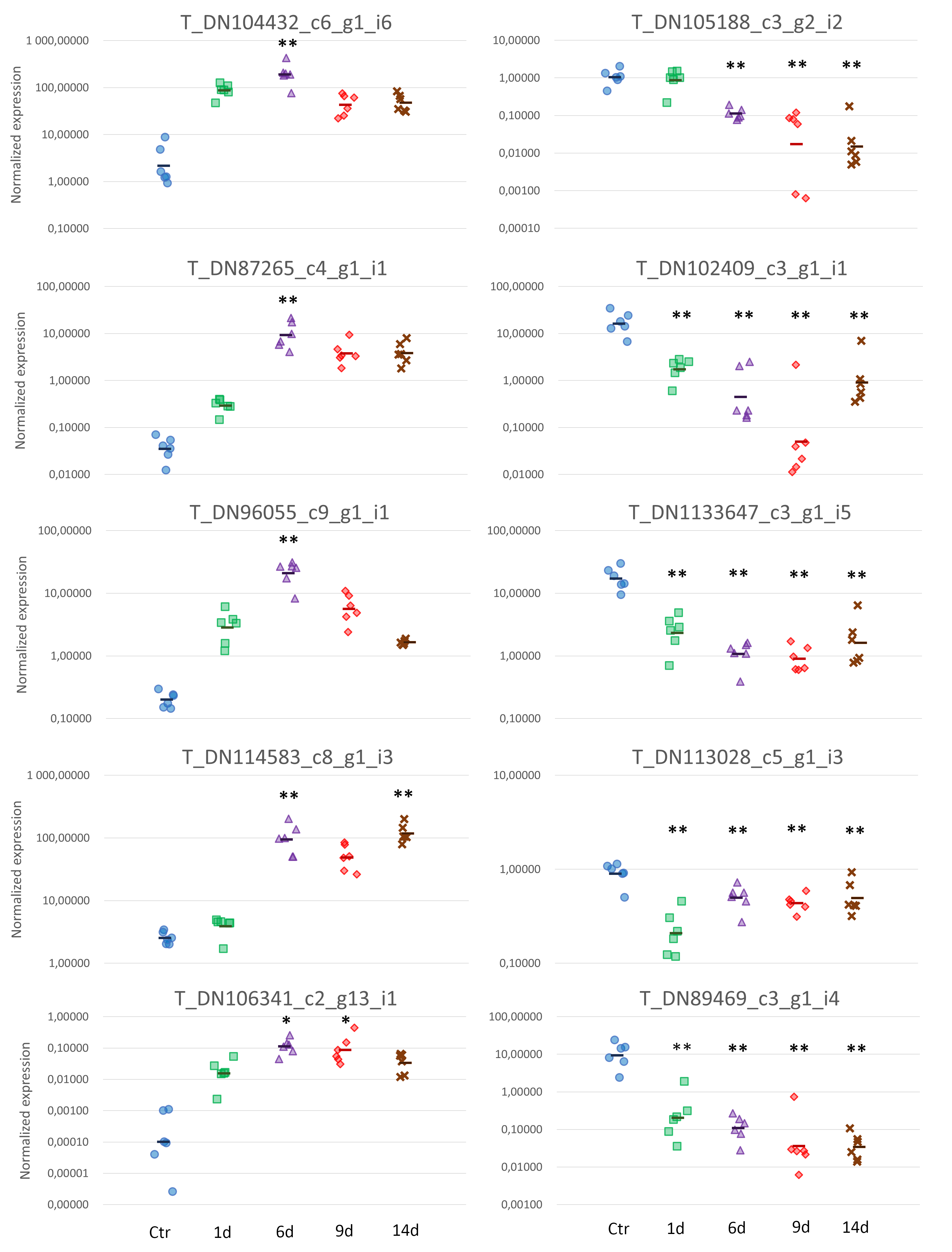

Supplement: Supplementary file 1 [file ijms-22-00941-s001.zip › Figure_S4_v2.tif]

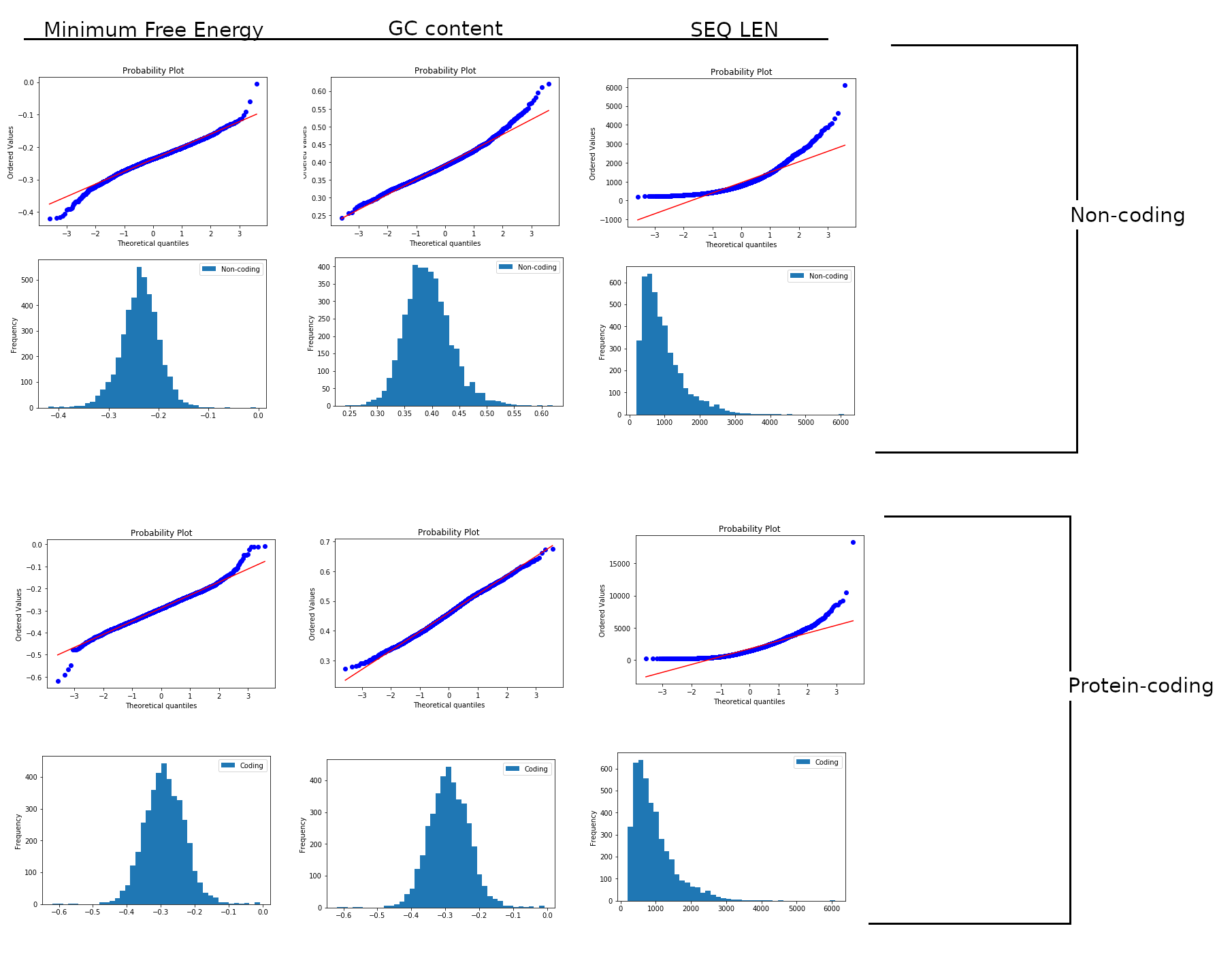

Supplement: Supplementary file 1 [file ijms-22-00941-s001.zip › Figure_S5_v2.tif]
