# Supplementary material for: De Novo Profiling of Long Non-Coding RNAs Involved in MC-LR-Induced Liver Injury in Whitefish: Discovery and Perspectives
Source: Int J Mol Sci. 2021 Jan 19;22(2):941. doi: 10.3390/ijms22020941 (PMC7833382; doi:10.3390/ijms22020941)
Supplement: Supplementary file 1 [file ijms-22-00941-s001.zip › Figure_S1.doc]

```
Figure S1. Alignment of our putative MALAT1 transcript to the seed sequence of human MALAT1 deposited in Rfam database. (A) Output of cmscan hit alignment. (B) ClustalO alignment.


A)

Hit alignments:
>> MALAT1  Metastasis associated lung adenocarcinoma transcript 1
 rank     E-value  score  bias mdl mdl from   mdl to       seq from      seq to       acc trunc   gc
 ----   --------- ------ ----- --- -------- --------    ----------- -----------      ---- ----- ----
  (1) !    0.0019   20.2   0.6 hmm       20       67 ..         343         391 + .. 0.79     - 0.24

                                ::::::::::::::.:::::::::::::::::::::::::::::::::: CS
                     MALAT1  20 aCAAUGAAGAucaA.AUgUCCAUCUCaAAAUaCUGCUUUUACAAAAGCA 67
                                a AAUGAA A      U U C UC CaAAA aCU CUUUUACAAAAGCA
  TRINITY_DN98182_c6_g2_i11 343 AAAAUGAAUACAUUuUUCUUCCUCACAAAAAACUACUUUUACAAAAGCA 391
                                4567888766655435567788999**********************98 PP


B)

TRINITY_DN98182_c6_g2_i11       AACAAACAGACGACCAAACAAUACGAUUCUGAUUCUGUAUACAAAAUGAAUACAUUUUUC    360
AAFR03057539.1/2772-2674        ----------UUAUGAAC------------GCUAUUUUAUUUGCAAUGAAGAGAGAAAUU    38
AAPN01356548.1/1259-1360        ACAAAAUUUAUGAGAA---------------AAUACCUAUUUCCAAUGAAGAGAAAGUAU    47
AAKN02038533.1/90323-90414      AAGGAAUUGAUGAGGA--------------------------ACAACGAAGAGCAAAUGU    35
AAFC03015964.1/24262-24162      AAUGAAUUGAUGAAAAAU------------AAAAAUAACGAUACAAUGAAGAUCAAAAUG    49
AAGV020417860.1/1991-1901       --UGAAUUGAUGAAAC---------------------ACUUAAAAAUGAAGAUCAAAUGU    37
AAEX03011638.1/22240-22150      AAUGAAUUGAUGAGAA------------------------AUAACAACGAAGAUCAAAUA    37
AAGW02079994.1/5712-5616        AAGGAAUUGAUGAGAA------------------------UUGAA-UGAAGAUCAAAUAU    36
ABRT010445981.1/4276-4184       AAUGAAUUGAUGAGAA------------------------AUGCAAUGAAGAUCAGAUGU    37
ABDC01174332.1/5507-5597        AAUGAAUUGAUGAGAA------------------------AUACAGUGAAGAUCAAAUGU    37
AAQR03147648.1/19088-19179      AAUGAAUUGAUGAGAG------------------------AUACAGUGAAGAUGAAAUGU    37
AAWR02037822.1/3627-3723        AACGAAUUGAUGAGAA------------------------AUACAAUGAAGAUCAAAUGU    37
ABRP01112167.1/2069-1975        AAUGAAUUGAUGAGCA------------------------AUACAAUGAAGAUCAAAUGU    37
ABGA01013318.1/1214-1126        ----UAAUGAUGAGAA------------------------AUACAAUGAAGAGCCAAUGU    32
FJ209305.1/3870-3963            AAUGAAUUGAUGAGAA------------------------AUACAAUGAAGAGGCAAUGU    37
AANU01213847.1/4092-3999        AAUGAAUUGAUGAGAA------------------------AUACAAUGAAGAGCCAAUGU    37
AACZ03082520.1/19366-19459      AAUGAAUUGAUGAGAA------------------------AUACAAUGAAGAGCCAAUGU    37
CABD02064759.1/1698-1791        AAUGAAUUGAUGAGAA------------------------AUACAAUGAAGAGCCAAUGU    37
                                            *                                    *


TRINITY_DN98182_c6_g2_i11       UUCCUCACAAAAAACUACUUUUACAAAAGCAUGGCAUCCUCACAUACACCCAGAGUACAU    420
AAFR03057539.1/2772-2674        U-CACCUCGAAAUGCUGCUUUUACAAAAGCAGAAUGAUUAAAAGAGAGAAAAAGGAAAAU    97
AAPN01356548.1/1259-1360        C-CAUCUCCAAAUGCUGCUUUUACAAAGGCAGAGAAAAAGCGCACACUGAAAAUCU----    102
AAKN02038533.1/90323-90414      CC-ACCACAAACCCUGCUUUUUACAAAAGCAAAAUAAAGUGAAAUGA---AACAAAAAUG    91
AAFC03015964.1/24262-24162      U--CAAUCUCAAUACUGCUUUUACAAAAGCAGAAUUAAAAAAGCGAAAUGAAAA------    101
AAGV020417860.1/1991-1901       C--CAUCUCAAAUACUGCUUUUACAAAAGCAGAAUAUAAAAGAAAAAUGAAAAUGA----    91
AAEX03011638.1/22240-22150      ACCAUCUCAAAAUACUGCUUUUACAAAAGCAGAAUAAAACAAAAUGAAAAAUGG------    91
AAGW02079994.1/5712-5616        UCAUCUCAAAAAUGCUGCUUUUACAAAAGCAGAAUAAAAAAGCGAAAUGAAAUGCAAAUG    96
ABRT010445981.1/4276-4184       CC-AUCUCAAAACUGCU--UUUACAAAAGCAGAAUUUUAAAAAGAUG--AAAUGAAAAUG    92
ABDC01174332.1/5507-5597        CC-GUCACAAAAUACUGCUUUUAGAAAAGCAGAAUUUAAAGAAAAAUGAAAAUCA-----    91
AAQR03147648.1/19088-19179      CC-GUCACAAAAUACUGCUUUUACAAAAGCAGAAUAAAAGAAAAGAAAUGAAAAAC----    92
AAWR02037822.1/3627-3723        CC-AUCUCCAAUUACUGCUUUUACAAAAGCAGAAUAAAAAAGCGAAAUGAAAUGAAAAUG    96
ABRP01112167.1/2069-1975        CC-AUCUCAAAAUACUGCUUUUACAAAAGCAGAAUAUAAGUGAAAU--GAAAUGAAAAUG    94
ABGA01013318.1/1214-1126        CC-AUCUCAAAAUACUGCUUUUACAAAAGCAGAAUAAAAGCGAAAU--GAAAUGAAAAUG    89
FJ209305.1/3870-3963            CC-AUCUCAAAAUACUGCUUUUACAAAAGCAGAAUAAAAGCGAAAA--GAAAUGAAAAUG    94
AANU01213847.1/4092-3999        CC-AUCUCAAAAUACUGCUUUUACAAAAGCAGAAUAAAAGCGAAAU--GAAAUGAAAAUC    94
AACZ03082520.1/19366-19459      CC-AUCUCAAAAUACUGCUUUUACAAAAGCAGAAUAAAAGCGAAAU--GAAAUGAAAAUG    94
CABD02064759.1/1698-1791        CC-AUCUCAAAAUACUGCUUUUACAAAAGCAGAAUAAAAGCGAAAU--GAAAUGAAAAUG    94
                                          *           * *** ***

TRINITY_DN98182_c6_g2_i11       CAUUGACACAAAUACCUUUGAUGGGCACCAAAAAUAACUUUAAAUGCAUCACAAAUUAAU    480
AAFR03057539.1/2772-2674        GC----------------------------------------------------------    99
AAPN01356548.1/1259-1360        ------------------------------------------------------------    102
AAKN02038533.1/90323-90414      A-----------------------------------------------------------    92
AAFC03015964.1/24262-24162      ------------------------------------------------------------    101
AAGV020417860.1/1991-1901       ------------------------------------------------------------    91
AAEX03011638.1/22240-22150      ------------------------------------------------------------    91
AAGW02079994.1/5712-5616        A-----------------------------------------------------------    97
ABRT010445981.1/4276-4184       A-----------------------------------------------------------    93
ABDC01174332.1/5507-5597        ------------------------------------------------------------    91
AAQR03147648.1/19088-19179      ------------------------------------------------------------    92
AAWR02037822.1/3627-3723        A-----------------------------------------------------------    97
ABRP01112167.1/2069-1975        A-----------------------------------------------------------    95
ABGA01013318.1/1214-1126        ------------------------------------------------------------    89
FJ209305.1/3870-3963            ------------------------------------------------------------    94
AANU01213847.1/4092-3999        ------------------------------------------------------------    94
AACZ03082520.1/19366-19459      ------------------------------------------------------------    94
CABD02064759.1/1698-1791        ------------------------------------------------------------    94
```

| Sequence ID | Species |
| --- | --- |
| AAFR03057539.1/2772-2674 | Pteropus vampyrus (large flying fox) |
| AAPN01356548.1/1259-1360 | Dasypus novemcinctus (nine-banded armadillo) |
| AAKN02038533.1/90323-90414 | Pongo abelii (Sumatran orangutan) |
| AAFC03015964.1/24262-24162 | Tarsius syrichta (Philippine tarsier) |
| AAGV020417860.1/1991-1901 | Bos taurus (cattle) |
| AAEX03011638.1/22240-22150 | Monodelphis domestica (gray short-tailed opossum) |
| AAGW02079994.1/5712-5616 | Ornithorhynchus anatinus (platypus) |
| ABRT010445981.1/4276-4184 | Microcebus murinus (gray mouse lemur) |
| ABDC01174332.1/5507-5597 | Equus caballus (horse) |
| AAQR03147648.1/19088-19179 | Homo sapiens (human) |
| AAWR02037822.1/3627-3723 | Cavia porcellus (Domestic guinea pig) |
| ABRP01112167.1/2069-1975 | Macaca mulatta (Rhesus monkey) |
| ABGA01013318.1/1214-1126 | Canis lupus familiaris (dog) |
| FJ209305.1/3870-3963 | Pan troglodytes (chimpanzee) |
| AANU01213847.1/4092-3999 | Gorilla gorilla gorilla (western lowland gorilla) |
| AACZ03082520.1/19366-19459 | Oryctolagus cuniculus (rabbit) |
| CABD02064759.1/1698-1791 | Otolemur garnettii (small-eared galago) |
