# Supplementary material for: De Novo Profiling of Long Non-Coding RNAs Involved in MC-LR-Induced Liver Injury in Whitefish: Discovery and Perspectives
Source: Int J Mol Sci. 2021 Jan 19;22(2):941. doi: 10.3390/ijms22020941 (PMC7833382; doi:10.3390/ijms22020941)
Supplement: Supplementary file 1 [file ijms-22-00941-s001.zip › Table_S2.doc]

**Table S2**. Contig metrics of filtered *de novo* assembled liver transcriptome. All metrics were calculated using Transrate, version 1.0.3.

| Contig | metrics |
| --- | --- |
| N seqs  (the number of contigs in the assembly) | 420280 |
| Smallest  (the size of the smallest contig) | 201 |
| Largest  (the size of the largest contig) | 17408 |
| N bases  (the number of bases included in the assembly) | 249597893 |
| Mean len  (the mean length of the contigs) | 593.88 |
| N under 200  (the number of contigs shorter than 200 bases) | 0 |
| N over 1k  (the number of contigs greater than 1,000 bases long) | 56150 |
| N over 10k  (the number of contigs greater than 10,000 bases long) | 30 |
| N with ORF  (the number of contigs that had an open reading frame) | 50256 |
| Mean ORF percent  (for contigs with an ORF, the mean % of the contig covered by the ORF) | 50.84 |
| N90  (the largest contig size at which at least 90% of bases are contained in contigs at least this length) | 274 |
| N70  (the largest contig size at which at least 70% of bases are contained in contigs at least this length) | 414 |
| N50  (the largest contig size at which at least 50% of bases are contained in contigs at least this length) | 776 |
| N30  (the largest contig size at which at least 30% of bases are contained in contigs at least this length) | 1542 |
| N10  (the largest contig size at which at least 10% of bases are contained in contigs at least this length) | 3030 |
| GC  (% of bases that are G or C) | 0.45 |
| Bases N  (the number of bases that are N) | 0 |
| Proportion N  (the proportion of bases that are N) | 0.0 |
