# Supplementary material for: De Novo Profiling of Long Non-Coding RNAs Involved in MC-LR-Induced Liver Injury in Whitefish: Discovery and Perspectives
Source: Int J Mol Sci. 2021 Jan 19;22(2):941. doi: 10.3390/ijms22020941 (PMC7833382; doi:10.3390/ijms22020941)
Supplement: Supplementary file 1 [file ijms-22-00941-s001.zip › Table_S3.doc]

**Table S3.** Sequences of primers used in RT qPCR.

| Name | Primer sequence [5’-3’] | Product length (bp) |
| --- | --- | --- |
| MALAT1 | F: GAATGGTTCCCCTGACATGGA  R: ATGTATACTAGACAGGGCAAGGT | 96 |
| HOTAIR | F: ACTGAGGGTTTTATGTGGGAAGA  R: TGCGACCCAATAGACGACAG | 137 |
| HOTTIP | F: AATGGCCTGCAAGTAAGAGACA  R: CGCCAGCTGAAAATAGAGCC | 164 |
| TRINITY_DN114583_c8_g1_i3  novel | F: CAGCCTCGTCCAGTTAGCAA  R: ACTGGGATAACCTTCCAAAATGA | 125 |
| TRINITY_DN87265_c4_g1_i1  novel | F: CGAATAGGGAGGCAATGCAAG  R: CTACCCCTGCACTCTGAGTTTT | 161 |
| TRINITY_DN104432_c6_g1_i6  novel | F: TTCAACACTATCACACCTAGAGCC  R: TACCTACTGCACTGCCATCG | 110 |
| TRINITY_DN96055_c9_g1_i1  novel | F: TTCCCCAAATTCCCTGGTTGG  R: CTGTCTCCACTAGGGTTGCAC | 133 |
| TRINITY_DN106341_c2_g13_i1  novel | F: AGTGAATTCCTGCTATGTGCCT  R: TCAGCTGACCACTGTGTTTT | 129 |
| TRINITY_DN105188_c3_g2_i2  novel | F: CATGTTGCCCCAAAAAGCCT  R: CATCGAACATGGACATCAAAGGT | 136 |
| TRINITY_DN102409_c3_g1_i1  novel | F: AGCAAACCCCCAAAGACACG  R: GGTGGTAGGAGATGAGGGTT | 160 |
| TRINITY_DN113028_c5_g1_i3  novel | F: AGTTGGGGCTTTATGGGCTG  R: TGGCAACTGGAAGCCCTATG | 115 |
| TRINITY_DN113647_c3_g1_i5  novel | F: ATTAGGTGTGGTCTGTCGC  R: AGCTGACCACCGATTTTCATTG | 138 |
| TRINITY_DN89469_c3_g1_i4  novel | F: TGACACCTCTCTCACTATTGTTT  R: ACTCGGCTCTGTCCAATAACT | 124 |
| TRINITY_DN96917_c3_g1_i1  reference | F: CTTCCTCCCGTATTGCTCCA  R: TGCCATTAGAGCCCCCATCT |  |
